# Supplementary material for: Are carbon emissions trading and green financial instruments synergistic? -Comprehensive quantitative research based on content analysis
Source: PLoS One. 2024 Mar 7;19(3):e0298601. doi: 10.1371/journal.pone.0298601 (PMC10919601; doi:10.1371/journal.pone.0298601)
Supplement: S1 Data — (ZIP) [file pone.0298601.s001.zip › Data/地级市Z_F工作报告.zip!/╣π╢1⁄2/╘╞╕í╩╨/└┤╫╘╣1⁄2╓┌║┼í╛┬φ┐╦╩2╛▌═°í┐_2017.docx]

各位代表：

我代表市人民政府向大会作工作报告，请予审议，并请政协委员和其他列席人员提出意见。

一、过去五年工作回顾

本届政府任期的五年，在省委、省政府和市委的正确领导下，在市人大及其常委会和市政协的监督支持下，坚决贯彻省委、省政府进一步促进粤东西北地区振兴发展战略和创新驱动发展战略等决策部署，紧紧围绕全面建设现代生态城市目标，坚持生态立市、产业兴市、特色美市、改革活市、依法治市，狠抓“招商、建城、改革”，基本完成“十二五”规划主要目标任务，实现“十三五”良好开局。

——坚持发展第一要务，经济发展稳中提质。

**发展速度持续较快。**初步预计，2016年，全市实现地区生产总值770.7亿元，同比增长7.9%，年均增长（下同）10.6%。地方一般公共预算收入57.4亿元，年均增长14.02%。规模以上工业总产值1200亿元，年均增长18.5%；规模以上工业增加值280亿元，年均增长16.9%。完成固定资产投资五年累计3224亿元，年均增长21.8%。外贸进出口总值五年累计完成87.52亿美元，年均增长8.3%。建设重大项目五年累计478个，完成投资1132亿元。实体经济进一步壮大，规模以上企业发展到1525家，比2012年增加722家。金融业快速健康发展。

**发展质量不断提高。**三次产业比重由2012年的23.6∶39.8∶36.6调整到20.3∶42.0∶37.7。社会消费品零售总额达344亿元，是2012年的1.9倍。新增专利申请3015件，年均增长30.7%；高新技术企业增至23家。工业技术改造投资五年累计完成85.35亿元，年均增长40.6%。实现高技术制造业增加值21亿元，年均增长18.6%；先进制造业增加值54亿元，年均增长17.2%。政府质量工作考核排全省前列。

——坚持优化经济结构，新兴产业发展初显成效。

**现代产业培育加快。**云计算及信息服务业发展提速，成功引进华为、神州数码等知名IT企业。先进装备制造业有新突破，与佛山飞驰汽车有限公司合作，引进加拿大先进大功率氢燃料电池电堆技术。市健康医药产业园、罗定中药提取产业基地建设加快，培育一力罗定制药、雷允上药业等规模以上医药企业11家。新兴县成为全国休闲农业与乡村旅游示范县，郁南县连滩镇西坝兰寨村、罗定市苹塘镇稻田景观分别被誉为“中国最美休闲乡村”、“中国美丽田园”。

**特色农业集优成势。**创建全国农产品质量安全市，创建省级以上农业标准化示范区37个，建设现代标准农田55.22万亩。粮食生产实现五连增，罗定市被评为全国粮食生产先进单位。温氏集团跻身世界农牧企业前三名，实现整体上市。培育国家农业产业化示范基地1个，全国一村一品示范镇1个、示范村4个；国家级驰名农产品商标1个，地理标志产品14个。云安、新兴、罗定、郁南的小型农田水利重点县建设进展顺利。累计完成水利投资33.28亿元，是上五年的2.3倍。

**工业载体建设加快。**工业园区由2012年的2个发展到“两园六区^①^”，面积达到52.4平方公里。建成佛山（云浮）产业转移工业园、佛山顺德（新兴新成）产业转移工业园两个省级产业园。累计获得园区扩能增效专项扶持资金6.61亿元，投入园区基础建设资金101.6亿元；入园项目406个，实现园区规模以上工业总产值375亿元，年均增长33.8%。两个省级产业转移工业园均获省考核优秀等次。帮扶成效显著，佛山市帮扶资金总规模达16亿元，引入项目341个，计划总投资330.8亿元。粤桂黔高铁经济带合作试验区广东园云浮分园获批。

——坚持交通先行，基础保障能力全面增强。

累计完成交通建设投入340亿元，是“十一五”时期的1.76倍。实现“县县通高速、县县通铁路”目标。南广铁路开通运行，云罗、江罗、罗阳等高速公路投入运营。清云、云湛、高恩、怀阳等高速公路建设加快。铁路运营里程达258公里，新增高速铁路105公里；高速公路通车总里程达300.6公里，五年新增182公里。新改建农村公路 2850公里。实现100%镇（街）有客运站、符合条件行政村100%通客车和有候车亭目标。顺利创建西江航道界首至南江口的全国文明样板航道，至肇庆3000吨航道扩能升级加快推进。与佛山等市联合推动珠三角枢纽机场前期工作。建成500千伏卧龙输变电项目等一批重点电力工程，建设改造了西江水厂管网等一批供水工程，光纤和移动宽带覆盖面不断扩大，供电、供水、通信能力大幅提高。

——坚持绿色发展，生态环境持续优化。

建成生态公益林304.37万亩，森林覆盖率达69.6%。农村生活垃圾处理工作连续三年（2014～2016年）在全省第三方评估考核中排粤东西北地区12市第一名。完成112条村生活污水处理设施建设。郁南县被国家确定为全国农村生活污水处理示范县。市区生活垃圾无害化处理率100%。饮用水源水质和西江云浮段水质均保持二类标准，集中式饮用水源和省控断面水质达标率100%。全年空气质量优良率达92.2%，是全省唯一连续三年（2014～2016年）全面达标的内陆城市。严格落实环评和“三同时”制度，全面完成1000个减排项目。国土资源管理水平不断提高，连续16年实现耕地占补平衡。

——坚持科学有序扩容提质，城乡建设统筹推进。

云安撤县设区。云浮新区管委会获省政府批准成立，《云浮新区发展总体规划》获省政府批准。“一核三副^②^”城市格局建设全面推进，“一江四组团^③^”架构基本形成。中心城区翠丰路等10条道路改造贯通。实现了国家卫生城市、广东省文明城市、广东省园林城市“三城联创”目标。罗定、新兴、郁南三个副中心扩容提质加快推进。罗定市建成环市路，新兴县建成二环路，郁南县建成高铁大道、九星新城等一批项目，拉开城区架构，城市承载力不断提高。市“一张蓝图”工程、罗定市、郁南县平台镇被列为全省新型城镇化综合试点。建成广东名镇7个、名村40条，创建生态文明村6053条。

——坚持民生优先，社会事业加快发展。

办好每年“十件民生实事”，累计总投资达149.85亿元。基本公共服务支出占财政一般预算支出比重由2012年的38.9%提高到2016年的49.72%。

**扶贫开发工作扎实推进。**圆满完成第一、二轮扶贫开发“双到”工作任务，累计投入帮扶资金12.2亿元，在省考核验收中均获优秀等次。开工建设各类保障性安居工程8211套（户），完成农村危旧房改造20878户。新时期精准扶贫投入帮扶资金1.07亿元，到村项目696个、到户项目3752个。

**卫生计生服务能力明显增强。**新建、改造医院12家。市人民医院新院建成投入使用，市中医院、妇幼保健院、慢病中心整体搬迁。成功创建4家“三级甲等”医院，在全省地级市率先创建全国基层（社区和农村）中医药工作先进单位。县级公立医院综合改革覆盖率100%。市人民医院创建成全国综合医院中医药工作示范单位。连续四年获广东省重性精神疾病管理治疗工作优秀市称号。全面二孩政策稳妥实施，人口计划生育服务管理水平不断提高。

**文化教育体育事业稳步发展。**市县镇村四级公共文化设施实现全覆盖。广播电视综合人口覆盖率100%。省级石艺文化创意产业园和禅文化创意产业园落户云浮。“扫黄打非”、文化执法持续常态化开展。罗定市、新兴县被评为全国文化先进县，郁南磨刀山遗址与南江旧石器遗址群入选“2014年度全国十大考古新发现”，新兴县图书馆被评为全国全民阅读先进单位。教育强县（市、区）覆盖率100%，成功创建广东省教育强市，五个县（市、区）创建成为国家义务教育发展基本均衡县。市中专学校、市高级技工学校、华立技师学院、伊顿实验学校建成使用。镇级农民体育健身广场实现全覆盖，成功举办第五届市运会。荣获全国残疾人体育先进单位称号。

**社会保障和就业工作成效显著。**认真落实“大众创业，万众创新”各项政策。人力资源社会保障基层服务平台实现全覆盖，新增城镇就业16.49万人，城镇登记失业率控制在3%以内。人才总量达26.93万人，比“十一五”期末增长28.9%。企业职工最低工资标准由每月710元提高到1210元，年均增长11.3%。残疾人服务设施覆盖率100%。

**平安云浮建设稳步推进。**公安执法规范化建设通过“四个一律^④^”专项检查，成为全省获得优秀等次的6个地级市之一。“民警+社工”戒毒社会工作新模式在全省推广。安全生产和食品药品安全形势持续稳定。国防动员体系不断完善，双拥工作取得新成绩。

——坚持改革创新，体制机制焕发新活力。

调整压减行政审批事项2039项，缩减审批环节589个。市县两级33508项权责事项全面公开。“无水港”通关模式在全省率先推行。“一门式、一网式^⑤^”政府服务模式改革加快推进，新兴县基层公共服务平台建设试点经验在全省推广。网上办事大厅开通服务，市公共资源交易中心正式运行。“1+10”创新驱动政策体系^⑥^、“1+3”人才政策体系^⑦^建立完善。创新设立“四平台五基金^⑧^”，130家企业在区域性股权交易市场注册、挂牌，普惠金融试点县（市）全部通过省考核验收。城乡一体化户籍制度改革全面实施。建设基层协同共治体系示范点25个。“六五”普法工作圆满完成。实现一村（社区）一法律顾问全覆盖目标。农村综合改革深入推进，突出“五个一^⑨^”改革重点，促进农村新发展。

——坚持依法行政，法治政府建设全面推进。

坚持向市人大及其常委会报告工作，向市人民政协通报情况，办理人大代表建议470件，政协提案899件。办理行政复议案件216宗。深入开展党的群众路线教育实践活动、“三严三实”专题教育及“两学一做”学习教育，严格执行中央八项规定，坚决纠正“四风”。“三公”经费和会议费支出连续五年实现“零增长”，比2012年下降61.3%。省依法行政考核由原来排位靠后跃升至2015年的第七名。

同时，民宗、侨务、港澳台、人防、统计、地震、地方志、消防、档案、保密、科协、侨联、文联、社科联、工商联、红十字、贸促、代建、志愿、社工、慈善、老龄以及驻市外机构等工作也取得新成绩。

各位代表，2016年是本届政府任期的最后一年。面对经济下行压力增大，市政府努力稳增长、调结构、促改革、惠民生，经济社会各项工作取得新成效。交通基础设施进一步完善。江罗、罗阳高速建成通车，新城快线南延段动工建设。扩容提质有新成效。云浮新区中央商务区城市综合体、西江新城绿化景观工程全面铺开。云城区田心片区综合改造动工建设。“四新一特^⑩^”产业加快发展。云计算大数据产业园动工建设。智慧云浮上线运营。飞驰氢能源整装客车实现量产，氢能源城市公交车在佛山、云浮两市示范运营。重点项目建设扎实推进。完成41个省级、83个市级重大项目年度建设任务，分别为115.47%和107.84%。创新能力进一步提升。佛山（云浮）氢能产业与新材料发展研究院成为省级新型研发机构。云浮创新设计中心、专业镇协同创新中心、广东工业大学云浮高新技术产业开发区博士后创新实践基地挂牌成立。新增国家级和省级创新平台8个。广东通力定造股份有限公司、广东惠云钛业股份有限公司在新三板上市。民生事业蓬勃发展。2016年民生类支出124.48亿元，占全市地方一般公共预算支出的72.4%。云浮第一所本科院校广东药科大学云浮校区启动建设。“十件民生实事”基本完成，一大批事关人民群众切身利益的就业、社保、住房、医疗卫生等重点难点热点问题得到解决。

五年的成绩令人瞩目，这是省委、省政府和市委正确领导、科学决策的结果，是全市人民齐心协力、不懈奋斗的结果。在此，我代表市人民政府，向全市人民，向人大代表、政协委员，向所有关心支持云浮的同志们、朋友们，致以崇高敬意和衷心感谢!

在肯定成绩的同时，我们必须充分认识当前发展存在的困难和不足，主要是：发展不充分仍然是最突出问题。与全省同步全面建成小康社会的要求差距较大。发展短板依然比较突出。城乡基础设施还不完善；教育、卫生、文化等民生配套与群众的期待还有一定差距；环境保护和生态建设有待加强。城市竞争力不强。城镇化发展的产业要素仍需进一步激活，城市配套和管理水平有待进一步提高。对此，我们必须采取有力措施加以解决。

二、今后五年的目标任务

未来五年，是云浮全面完成“十三五”规划目标任务，全面建成小康社会的关键和决胜时期。我们要紧紧把握机遇，坚持科学发展，奋力后发赶超，谋求跨越发展。

今后五年政府工作的总体要求是：全面贯彻党的十八大和十八届三中、四中、五中、六中全会精神，深入贯彻习近平总书记治国理政新理念新思想新战略，统筹推进“五位一体^⑪^”总体布局和协调推进“四个全面^⑫^”战略布局，牢固树立创新、协调、绿色、开放、共享的发展理念，适应把握引领经济发展新常态，坚持稳中求进工作总基调，以推进供给侧结构性改革为主线，按照市委“生态立市、产业兴市、特色美市、改革活市、依法治市”和“一同步六提升^⑬^”总体要求，狠抓“招商、建城、改革”三大任务，突出发展经济、保障民生两大重点，全面加快建设现代生态城市，与全省同步全面建成小康社会。

经济社会发展主要目标是：到2018年，与全省同步全面建成小康社会，全面小康综合指数达97%以上，生产总值和城乡居民人均收入比2010年翻一番以上。到2021年，地区生产总值突破1300亿元；工业总产值突破2000亿元，其中以“四新一特”为重点的新产业总值形成1000亿元规模，现代产业新体系基本形成；地方一般公共预算收入年均增长10%；固定资产投资年均增长12%；城乡居民人均可支配收入年均增长12%；社会消费品零售总额年均增长10%。相对贫困人口实现全部脱贫。

——强化生态建设，提升环境竞争力。严格保护原生态、积极修复病生态、主动建设新生态，守住生态优势。大力发展循环经济，抓好节能减排，推进资源节约集约利用。深入实施大气、水、土壤污染防治行动计划，统筹推进城乡生活污水、生活垃圾、中小河流等综合治理。积极实施生态景观林、森林进城围城、中心城区增绿提质等生态工程，构建地绿水清景美的城市生态环境。加快城市园林绿化建设步伐，确保2019年通过国家园林城市考核验收。到2021年，空气优良率保持90%以上，森林覆盖率达70%以上，中心城区人均公园绿地面积达8.4平方米。

——强化产业建设，提升经济综合实力。谋划新产业，重点发展云计算大数据、生物医药、先进装备制造、健康养生旅游产业和现代特色农业。建设电商区域性总部基地、大数据处理产业基地、信息服务及外包产业基地和数字媒资产业基地，成为华南领先乃至全国有名的云计算大数据产业集聚地。加快广东药科大学云浮校区建设，创建中国南方中药资源中心、生物医药研究与开发基地、生物医药南方大数据中心等平台，到2021年实现年总产值超100亿元，成为华南地区重要的生物医药产业基地。推进氢能源汽车及汽配、石材装备、农牧装备、环保装备等产业集群发展，打造先进装备制造业区域合作示范基地。到2021年，集聚形成5个具有百亿产值的“珠江西岸装备制造产业转移先行区”。加快建设新兴禅文化健康养生度假区、罗定田园观光体验旅游区，规划建设云安云雾山旅游度假区、云城石文化休闲旅游区、大金山生态休闲公园、郁南北部生态旅游带^⑭^和南江文化历史风俗旅游区，打造全国知名的健康产业高地和休闲养生目的地。建设南药、稻米、果蔬、花卉苗木、生态养殖等特色产业基地，打造全国现代特色农业发展示范基地和珠三角健康食品生产基地。主动对接“中国制造2025”战略，深入实施“互联网+”和工业转型升级攻坚战行动计划，推动石材、不锈钢制品、硫化工等传统产业向产业链两端延展、向价值链高端提升。

——聚力创新驱动，提升发展质量。到2021年，建成一个国家级高新技术开发区，科技进步贡献率达60%以上；高新技术企业数量实现翻一番；引进10个以上总投资超10亿元的大项目；扶持4家企业成功在A股上市，10家以上企业成功在新三板上市，300家以上中小微企业在区域性股权交易市场挂牌。创建一批省级新型研发机构，抓好佛山（云浮）氢能产业与新材料发展研究院、市专业镇协同创新中心等建设。深入实施“六大人才引育工程^⑮^”，新增专业技术技能人才6万人。

——聚力交通建设，提升区位优势。抓好高速公路、航道、机场等外循环交通基础设施建设。到2021年，计划总投资410亿元，其中高速公路投资174亿元，水路、机场、铁路和客货运站场等投资69亿元。完成高速公路建设125公里，通车总里程突破420公里；新增铁路通车里程34公里，通车总里程达292公里。做好珠三角枢纽机场前期工作，升级改造云浮罗定机场。加快西江航道扩能升级。谋划肇云轻轨、广湛高铁、佛新云轻轨、柳广铁路、张家界经云浮至海口高铁旅游客专、西江大桥等建设。抓好市内交通和高速公路互通口连接线的内循环交通基础设施建设。投入167亿元建设改造国道、省道和农村公路2500公里。建成东部快线^⑯^、新城快线二期、西部快线^⑰^、南部干线^⑱^、广梧高速公路增设新区立交等一批交通路网。

——加快城市建设，提升宜居宜业水平。做好城市规划和医疗、教育配套设施建设，加快城市环境改造。加快推进基础设施、现代产业、城市公共服务、生态景观、创新平台、云安与新区一体化等六大建设工程。加快建设云浮新区核心区—西江新城，构建新城与各组团15分钟交通圈，新城人口规模达到13万人。推进老城区提质和新老城区融合发展，重点抓好田心片区、牧羊片区和城西片区的升级改造。统筹推进罗定、新兴、郁南三个副中心扩容提质。抓住珠江—西江经济带规划实施、粤桂黔战略合作等机遇，加快高铁经济带、高速公路经济带规划建设，把交通一体化、产业一体化、城市一体化、服务一体化、环保一体化作为合作对接的突破口，实现与广佛肇清韶地区融合发展。

——加快新农村建设，提升农村人居环境。推进农村电网建设和改造。加强农村饮用水安全，农村自来水普及率达85%以上。抓好省级新农村连片示范建设工程、省扶持村级集体经济发展试点、现代农业经营机制、农村土地“三权分置”改革、农村公共服务平台等工作，全面激发“三农”发展新活力。推进农村人居环境综合整治，创建10个省级和50个市级休闲农业与乡村旅游示范点。

——加强民生建设，提升群众幸福感。提高城乡基本公共服务均等化水平。高水平普及15年基础教育，到2018年，成为广东省推进教育现代化先进市；到2021年，实现学前教育毛入园率100%，公益普惠性学前教育服务体系全面构建；高中阶段教育毛入学率95%以上。建成广东药科大学云浮校区、恒大高级中学等13所学校。罗定职业技术学院、广东新兴中药学校、邓发纪念中学等学校扩容促优取得成效。基本建立现代公共文化服务体系,每万人拥有室内公共文化设施面积超过1200平方米。广播电视实现户户通。构建城乡公共卫生服务体系，到2020年，基本建成卫生强市和中医药强市；到2021年，实现卫生强县（市、区）覆盖率100%。卫生镇、卫生村普及率分别达50%、70%。计划生育服务管理水平不断增加。社会保障能力持续提升，基本社会保险覆盖率达98%以上，住房保障达到全省中上水平，城镇新增就业10万人，促进创业0.8万人，城镇登记失业率控制在4%以内。到2018年底，全市12.35万相对贫困人口实现脱贫、105个相对贫困村全部出列。

——加强政府建设，提升服务水平。持续推进简政放权、放管结合、优化服务，提高政府效能，增强政府公信力和执行力。稳妥推进机构改革和深化镇（街）行政体制改革。扎实推进政务公开。深入实施政府部门权责清单管理制度。推进“互联网+电子政务”体系建设，完善部门之间数据共享。深入构建平安云浮，营造安全稳定的社会环境和公平正义的法治环境。加强国防动员力量建设，拓展军民融合协调，建立新型国防动员体系。深化标本兼治，坚定不移惩治腐败，扎实推进廉政建设。坚持依法行政，构建职能科学、廉洁高效、阳光运作、人民满意的服务型政府。

三、2017年工作安排

2017年是实现与全省同步全面建成小康社会目标的关键之年，也是新一届政府工作的开局之年，做好今年工作意义重大。**经济社会发展主要预期目标是：**地区生产总值增长8.5%，地方一般公共预算收入增长8%，规模以上工业增加值增长10 %，固定资产投资增长9%，社会消费品零售总额增长11%，居民消费价格涨幅控制在2.5%以内，外贸进出口总额增长2%，城乡居民人均可支配收入与GDP增长同步，完成省下达的节能减排任务。安排116项重点建设项目，计划总投资1283.88亿元，年度计划投资194.87亿元。围绕上述目标，重点抓好八方面工作：

（一）牢牢守住生态优势。

深入实施森林碳汇、乡村绿化美化等重点生态工程，改造疏残林、纯松林和布局不合理的速生林，推进3000亩新老城区林相改造，实现森林面积、森林蓄积“双增”目标。继续抓好水污染防治行动和南粤水更清行动年度计划实施，重点推进市区应急备用水源、镇级以上饮用水源地保护、黑臭水体治理等工程建设。加强大气污染治理力度，大力治理违法违规石材、水泥、硫化工、陶瓷等行业企业。完成4715辆“黄标车”淘汰任务。大力推进畜禽养殖和农业面源污染防治。扎实开展环境污染第三方治理，实施严格的环境准入和环保“三同时”制度。对不达标排放的企业实行“零容忍”，全面提升大气、水、土壤等污染治理水平。全面推进企业“十三五”绿色清洁生产审核。

（二）全力加快现代产业建设。

**发展壮大“四新”产业。**大力推进云计算数据中心、服务外包及呼叫中心产业园建设，加快软通动力集团两化融合基地建设进程。继续深化智慧城市服务应用，提升智慧云浮平台服务功能。推进光纤入户、4G通信基站、WLAN热点等信息化工程。加快发展先进装备制造业。推动氢燃料电池客车整车规模化生产。争取建成国家级氢能及燃料电池实验室和氢能检测中心。引入更多氢能源领域企业，不断完善氢能产业链。争取省将我市纳入珠江西岸先进装备制造产业带。利用“互联网+智能制造”，提升石材、不锈钢制品、农牧等传统机械装备制造业水平。大力发展生物医药产业，加快市健康医药产业园、罗定中药提取基地、广东南药生产加工基地建设。加快发展健康养生旅游产业，推进中国新兴·禅宗文化产业基地等禅宗文化旅游和云雾山旅游度假区、永盛健康生态园等乡村休闲旅游项目。

**做优做强现代特色农业。**按照“四区两带^⑲^”农业发展新格局，积极实施农业主导产业提升、品质农业创建等“十大工程^⑳^”。加快建设现代农业生产基地、现代农业加工园区和农业主题公园。大力发展“互联网+现代农业”、“标准化+现代农业”，继续办好广东云浮·罗定稻米节等农业经贸活动。巩固国家农产品质量安全市创建成果，主要农产品总体合格率稳定在98%以上。

**转型升级传统产业。**加快建设完善国家石材检测中心、国家石材价格发布中心、国家石材大数据中心、国家石材交易中心。支持石材转型升级示范项目建设，推动石材产业往研发设计和营销方向转型升级。办好第十四届中国（云浮）国际石材科技展览会暨第八届中国（云浮）石文化节。深入开展不锈钢制品产业智能化生产技术改造，推动新兴县不锈钢餐厨具产业聚集区创建为知名品牌示范区。加大水泥产业节能环保技术改造，加快循环资源利用中心项目建设，完善循环经济产业链。深入开展硫酸生产线改造，提高硫化工产品附加值。

**全力推进产业共建。**大力实施佛山云浮产业共建行动计划，推动粤桂黔高铁经济带合作试验区广东园云浮分园建设。科学规划产业布局，推动两市一二三产业深度融合发展，强化民生社会事业领域协作共建。推动70个珠三角地区重点工业项目落户“两园六区”发展，实现共建产业园区规模以上工业增加值120亿元。争取15个以上大型优质项目落户产业共建园区核心区（思劳片区）。规划建设云浮新区—云安区产业园。推动海尔（罗定）工业园、凤铝工业园、国际现代农牧装备产业园、绿色日化产业集聚地、华润西江发电厂等项目早日动工或投产运营。

（三）深入实施创新驱动发展战略。

**大力推进创新要素集聚。**用足用活“1+3”人才新政，引进培养各类人才。加快建设一批新型研发机构，建成9家以上省级或市级工程技术研究中心和重点实验室，充分发挥市专业镇协同创新中心作用，发展壮大专业镇。重点培育云计算大数据、生物医药、氢能源等现代产业科技孵化中心。筹建广东药科大学云浮研究院、仲恺农业工程学院禅农研究院、广东省质量监督水泥和墙体材料检验站（云浮）以及广东省质量监督畜牧机械检验站（云浮）。支持企业建设工程技术研究中心（重点实验室）。培育高新技术企业5家以上。认定知识产权优势企业3家以上。加大名牌产品、著名商标、特色农产品商标、地理标志证明商标的培育。

**深入推进“三去一降一补”。**去产能优结构，重点跟踪53家特困企业，水泥熟料产能控制在1500万吨以内。去库存调供需，化解5万平方米商品房库存。去杠杆稳金融，银行业金融机构不良贷款占比与全省平均水平基本持平。全面深化普惠金融建设，推动“四平台五基金”与银行和保险机构全面对接，争取华夏银行进驻、罗定农信联社改制成为农商银行、广东温氏集团财务公司开业，以及广东新金山生物科技、罗定稻香园、广东十二岭酒业等企业登陆新三板。降成本促发展，落实电力大用户直购电政策。补短板增供给，投入167.2亿元推进35项补短板项目建设。

**提高招商选资实效。**坚持“四个指标，一个带动”，全面提升招商选资质量。紧紧围绕“四新一特”产业和传统产业强链补链，对国内外大型企业精准招商，举办产业专题招商推介会，力争引进大企业大项目100个以上，计划总投资超300亿元。完善“智慧招商”平台，深化“轻资产”招商引资模式。深化“无水港”通关模式，把云浮新港升级为全省“海上丝绸之路”的重要节点。

**扩大新消费新需求。**稳定房地产、汽车等大宗消费。大力推广电子商务，鼓励罗定“梦想小镇”等电商平台做优做强。建设“淘宝·特色中国·广东云浮馆”、“京东·中国特产·云浮馆”项目，争创省电子商务示范基地，支持创建省农村电子商务示范县。依托一批城市综合体打造城市消费新商圈，加快提升传统零售业。积极构建农村电商销售体系，建成罗定、新兴、郁南3个县级“农村电商”快递物流服务中心。

（四）全面加快基础设施建设。

**完善立体大交通体系。**计划投入交通基础设施建设资金65.6亿元。重点抓好汕湛高速公路清云段和新阳段、高恩高速公路新兴段、怀阳高速公路郁南段、罗信高速等项目建设，完成西江航道3000吨级扩能升级工程。升级改造罗定机场和南江口码头，推动珠三角枢纽机场规划选址。重启罗岑铁路建设。积极做好罗定至德庆高速公路北段、肇云轻轨、云浮新港疏港铁路等项目前期工作。谋划怀集至阳江西高速公路，争取尽快纳入省规划项目。抓好新城快线南延段、思劳立交东移项目建设，优化广梧高速公路云浮城区出入口连接线。推进东部快线、西部快线和新城快线二期建设。启动南部干线前期工作，力争年底动工建设。建设农村公路160公里，加快客运站升级改造和城乡客运发展，逐步实现城乡客运一体化。

**优化公共基础设施。**加快工业集中区、重点城镇的供排水、供电、供气、道路等公用基础设施建设。规划建设城市综合管廊6.4公里。城市燃气化率达99%以上。光纤入户率力争达71%。计划投入资金2.15亿元，完成年度12宗项目建设任务，治理中小河流107.2公里。全面改善城乡水利基础设施。继续推进全国国土资源节约集约模范县（市、区）创建活动。

（五）统筹加快新型城镇化建设。

**加快新区建设。**推动新区与云安区在城乡空间、土地利用、产业建设、生态保护等各个领域的规划衔接。突出重点，全力推进交通等基础设施、云计算及信息服务等现代产业、药科大学云浮校区等城市公共服务、北湖等生态景观、院校科研机构等创新平台、“两区”一体化等六大工程建设，加速城市要素集聚，整体提升环境吸引力，增强对产业的支持和服务，促进人口加快集聚。

**提升城市品位。**积极推进海绵城市和人文城市建设。加快城市地下管线普查和地下综合管廊试验段建设。加快田心片区综合改造、城区主干道升级和绿化景观改造，促进新老城区融合发展。继续实施城市道路贯通工程，重点抓好东方路、浩林西路、育才路、新城快线南延段、乐谊路东段等城区道路贯通改造。推进“三旧”改造项目建设。积极实施“六大提质工程”，创建成为国家园林城市。巩固国家卫生城市创建成果，确保通过复审。

**建设特色小镇。**按照“生产、生活、生态”融合的要求，依托各地资源禀赋和产业特色，规划建设云浮氢能产业特色小镇、禅文化产业特色小镇、云计算大数据产业（云谷）特色小镇、健康医药产业特色小镇以及南江文化、古驿道特色小镇。

（六）大力加快新农村建设。

**深化农村综合改革。**推进国家农产品质量安全市、全国农村改革试验区试验项目、新兴县国家农村三产融合发展试点示范县、罗定市国家农业可持续发展试验示范区建设。开展罗定、郁南全国农村“两权”抵押贷款试点。深入推进农业供给侧结构性改革，抓好现代农业经营机制、新农村连片示范建设工程、村级集体经济发展试点、农村环境综合治理等工作。围绕罗定稻米产业、新兴畜牧产业，积极推进开展政府购买农业公益性服务机制创新试点。抓好农村土地承包经营权确权登记颁证和农村集体“三资”管理工作。

**改善农村人居环境。**加快省级新农村示范片、生态文明示范镇、幸福村居和宜居镇、村、社区建设。积极创建南江口、连滩镇国家生态文明建设示范区。落实最严格水资源管理制度，水功能区水质达标率78%以上，巩固提升农村饮用水安全水平，农村自来水普及率85%以上。加大城区、中心镇污水处理和城乡垃圾无害化处理设施建设力度，城乡生活垃圾无害化处理率达85%以上，农村生活垃圾处理率达95%以上。完成100个以上农村生活污水治理工程项目。全面完成中心村农村电网改造升级任务。

（七）增进民生福祉。

**强化基本民生保障。**把扶贫攻坚与解决民生问题结合起来，扎实抓好精准扶贫、精准脱贫，确保4.25万相对贫困人口脱贫。推进全民参保计划，城乡居民养老保障基础养老金标准从每人每月110元提高到120元。稳步推进机关事业单位养老保险制度改革。提高企业退休人员基本养老金、城乡居民基本养老保险基础养老金和城乡居民医疗保险财政补助标准。扩大社保“一卡通”应用范围。扎实开展农村养老服务建设“幸福计划”。把市福利院、云安区福利服务中心、郁南县中心敬老院和罗定市区域性敬老院纳入全省第一批公建民营社会化改革试点。加快推进云城、新兴、云安富林镇区域性敬老院和市残疾人康复中心建设。帮扶1000名就业困难人口实现再就业。建立市县镇村四级复退军人服务机构，进一步完善复退军人服务体系。

**全力创建卫生强市。**加大设备设施投入，加快引进培养人才，大力推进医疗卫生强基创优工作。建设中医药强市。加快乡镇卫生院标准化和村卫生站公建民营规范化建设。从市、县层面，以人才引进方式招录200名临床医学大学毕业生充实到乡镇卫生机构。推动市人民医院区域医疗中心建设。推动市中医院成为广东药科大学直属附属医院。升级改造市妇幼保健院。做好迎接全国基层中医药工作先进单位复审工作。以城市公立医院综合改革为重点，继续深化医药卫生体制改革。进一步提高重性精神病管理治疗能力。稳步提升计划生育服务管理水平。

**大力发展教育事业。**全力加快广东药科大学云浮校区建设，争取首期建设完成封顶。大力支持罗定职业技术学院扩容提质。申请设立广东华立职业技术学院云浮校区。建成恒大高级中学，加快建设光明中学、光明小学。加快建设广东省教育现代化先进市，把新兴县率先创建成为广东省推进教育现代化先进县。发展学前教育和成人教育，设立云浮开放大学。完善职业教育体系，确保新兴县和郁南县通过省级农村职业与成人教育示范县验收。鼓励和规范民办教育。

**注重文化引领。**加快推进省第一、二批图书馆及文化馆总分馆试点、基层综合性文化服务中心示范点建设。在市区建设一批书吧和报刊杂志亭，提升城市文化品位。培育“百姓文化俱乐部”、“农村春晚”等文化活动品牌。探索设立国家级文化大师云浮工作室。弘扬优秀传统文化，加强文化遗产和非物质文化遗产保护。加大“扫黄打非”和文化执法力度。加快城乡体育场地设施建设。大力开展全民健身活动，促进群众体育、竞技体育和体育产业协调发展。

**全面推进平安云浮建设。**大力排查化解不稳定因素，维护社会和谐稳定。全面铺开镇（街）“中心+网格化+信息化”和以视频监控为重点的立体化社会治安防控体系建设。积极开展重点区域整治，实施全民禁毒工程。持续开展打击涉恐、涉黑恶以及各类突出违法犯罪专项行动，抓好互联网虚拟社会治安管控工作。强化食品药品和安全生产监管，严防发生重特大安全事故。严厉打击制售假冒伪劣商品违法行为。

（八）全面加强政府能力建设。

**大力推进简政放权。**推进企业投资项目“一门式、一网式”政府服务模式。加大政务公开工作力度。完善政府权力清单制度，健全政府效能督察机制。推进社会信用体系建设。推进以电子营业执照为核心的全程电子化登记管理改革，探索电子营业执照在行政审批、电子商务等领域的应用。建设应用“商事主体信息管理平台”，打造企业信用监管“全市一张网”。

**深入推进法治政府建设。**严格依法行政，完善重大行政决策咨询、绩效评估机制和终身责任追究制度、责任倒查机制。认真实施“七五”普法规划，加强对政府立法工作的统筹。完善人民调解与行政调解、司法调解联动工作体系，确保基层公证处建设达标率达70%以上，调解成功率和调解协议履行率保持98%以上。

**狠抓作风建设。**深入贯彻落实中央八项规定精神，坚决纠正“四风”。继续做好公车改革的后续工作和价费改革工作。坚持“三个区分”，激励干部干事创业。推广应用政府投资工程廉情预警评估系统。保持惩治腐败高压态势。深化以案治本工作，促进惩治成果向预防成果转化。推进监察体制改革，强化依法履职、秉公用权监督。

各位代表！回顾过去，我们奋力拼搏，发展步入了快车道；展望未来，我们任重道远，还须不懈努力。让我们在市委的坚强领导下，齐心协力，锐意创新，真抓实干，为加快建设现代生态城市，全面建成小康社会而努力奋斗！
